# Influence of microarrays experiments missing values on the stability of gene groups by hierarchical clustering

**DOI:** 10.1186/1471-2105-5-114

**Published:** 2004-08-23

**Authors:** Alexandre G de Brevern, Serge Hazout, Alain Malpertuy

**Affiliations:** 1 Equipe de Bioinformatique Génomique et Moléculaire (EBGM), INSERM E0346, Université Denis DIDEROT-Paris 7, case 7113, 2, place Jussieu, 75251 Paris Cedex 05, France; 2 Atragene Bioinformatics, 4 Rue Pierre Fontaine, 91000 Evry, France

## Abstract

**Background:**

Microarray technologies produced large amount of data. The hierarchical clustering is commonly used to identify clusters of co-expressed genes. However, microarray datasets often contain missing values (MVs) representing a major drawback for the use of the clustering methods. Usually the MVs are not treated, or replaced by zero or estimated by the k-Nearest Neighbor (*kNN*) approach. The topic of the paper is to study the stability of gene clusters, defined by various hierarchical clustering algorithms, of microarrays experiments including or not MVs.

**Results:**

In this study, we show that the MVs have important effects on the stability of the gene clusters. Moreover, the magnitude of the gene misallocations is depending on the aggregation algorithm. The most appropriate aggregation methods (e.g. complete-linkage and Ward) are highly sensitive to MVs, and surprisingly, for a very tiny proportion of MVs (e.g. 1%). In most of the case, the MVs must be replaced by expected values. The MVs replacement by the *kNN *approach clearly improves the identification of co-expressed gene clusters. Nevertheless, we observe that *kNN *approach is less suitable for the extreme values of gene expression.

**Conclusion:**

The presence of MVs (even at a low rate) is a major factor of gene cluster instability. In addition, the impact depends on the hierarchical clustering algorithm used. Some methods should be used carefully. Nevertheless, the *kNN *approach constitutes one efficient method for restoring the missing expression gene values, with a low error level. Our study highlights the need of statistical treatments in microarray data to avoid misinterpretation.

## Background

The genome projects have increased our knowledge of genomic sequences for several organisms. Taking advantage of this knowledge, the microarrays technologies allow the characterization of a whole-genome expression by showing the relative transcript levels of thousand of genes in one experiment [1]. Numerous applications were developed apart gene expression analysis like single nucleotide polymorphism (SNP) and genotyping [2,3], diagnosis [4] and comparative genomics [5] analysis. Particularly, the transcriptome analysis provides insight into gene regulations and functions. To help the characterization of relevant information in microarray data, specific computational tools are needed. The identification of co-expressed genes is commonly performed with unsupervised approaches, such as clustering methods with the Hierarchical Clustering (HC) [6], the *k*-means [7] and the Self-Organizing Map (SOM) [8,9], or with projection methods such as the Principal Component Analysis (PCA) [10] and Independent Component Analysis (ICA) [11].

Among these techniques, the HC approach is a widely held method to group genes sharing similar expression levels under different experimental conditions [12]. The HC is performed from the distance matrix between genes computed from the microarray data, *i.e*. the gene expression levels for various experimental conditions. Different aggregation methods can be used for the construction of the dendogram generally leading to different tree topologies and *a fortiori *to various cluster definitions [13]. For instance, the single-linkage algorithm is based to the concept of joining the two closest objects (*i.e*. genes) of two clusters to create a new cluster. Thus the single-linkage clusters contain numerous members and are branched in high-dimensional space. The resulting clusters are affected by the chaining phenomenon (*i.e*. the observations are added to the tail of the biggest cluster). In the complete-linkage algorithm, the distance between clusters is defined as the distance between the most distant pair of objects (*i.e*. genes). This method gives compact clusters. The average-linkage algorithm is based on the mean similarity of the observations to all the members of the cluster. Yeung and co-workers [14] showed that single-linkage hierarchical clustering is inappropriate to analyze microarray data. Gibbons and Roth [15] showed by using gene ontology that single-and average-linkage algorithms produce worse results than random. In addition these authors conclude that the complete-linkage method is the only appropriate HC method to analyze microarrays experiments.

The microarrays experiments frequently contain some missing values. The missing values are part of the experimental errors due to the spotting conditions (e.g. spotting buffer, temperature, relative humidity...) and hybridization (e.g. dust on the slide...) [16,17]. The users commonly discard suspicious dots during the images analysis step. Thus, the resulting data matrix contains missing values (MVs) which may disturb the gene clustering obtained by the classical clustering methods, e.g. HC, SOM, or projection methods, e.g. PCA. To limit the effects of MVs in the clustering analyses, different strategies have been proposed: (i) the genes containing MVs are removed, (ii) the MVs are replaced by a constant (usually zero), or (iii) the MVs are re-estimated on the basis of the whole gene expression data. Few estimation techniques have been applied to such data. The *k*-nearest neighbors approach (*kNN*) computes the estimated value from the *k *closest expression profiles among the dataset. Troyanskaya and co-workers showed that the weighted *kNN *approach, with *k *= 15, is the most accurate method to estimate MVs in microarray data compared to replacement by zero, row average, or Singular Value Decomposition [18]. A recent work proposes Bayesian principal component analysis to deal with MVs [19]. In the same way, Zhou and co-workers [20] have used a Bayesian gene selection to estimate the MVs with linear and non-linear regression. However, the *kNN *approach is the most popular approach for estimating the MVs.

To explore the incidence of MVs in gene clustering, we first assessed the proportion of MVs in different sets of public data from *Saccharomyces cerevisiae *and human. Observing that MVs are widely present in the expression data, we then analyzed the effects of MVs on the results of a hierarchical clustering (HC) according to the chosen clustering algorithm. In the same way, we evaluated the impact of MVs replacement and estimation in the gene cluster definition by using a hierarchical clustering method.

## Results and Discussion

### Missing values overview

Table 1 summarizes the proportions of MVs in eight series of microarray experiments [21-28]. The number of genes and experimental conditions are within the range (*552*; *16523*) and (*4*; *178*) respectively. The percentage of MVs varies from 0.8% to 10.6%. No relation had been found between the number of genes and the percentage of MVs or the percentage of genes without MVs. As expected, the percentage of genes with MVs increases in function of the number of experimental conditions. In the Sorlie [22], Spellman [28] and Gasch sets [25] respectively, 94.7%, 91.8% and 87.7% of the genes profiles have MVs. In these cases, it is not possible to systematically delete the genes profiles with MVs in the data analysis. Indeed, the percentage of MVs is not negligible in the microarrays data and may be a strong factor of gene clustering instability. Hence, we evaluated in this study the effects of MVs on gene group definition by using hierarchical clustering algorithms.

### Main steps of the analysis

**Figure 1:**
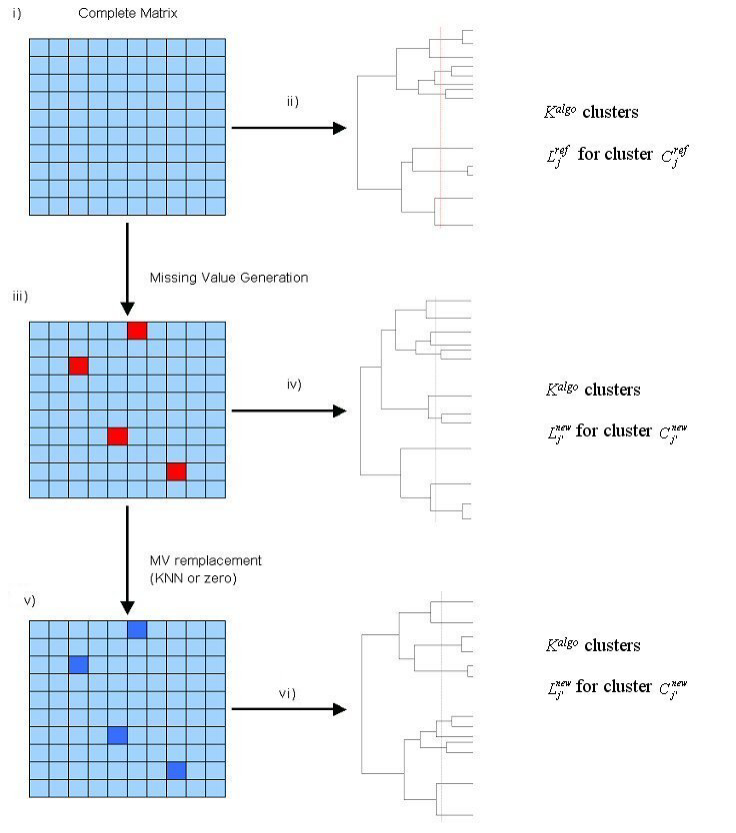
**Research of the co-associated genes in clusters. **(i) The original dataset is cleaned by removing all the genes with MVs. (ii) A hierarchical clustering is performed for a given clustering method and the 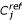 clusters are selected to characterized the original co-association of the genes, (iii) MVs are generated with a *τ *rate per gene, and replaced by zero or by an estimated value computed with the *kNN *approach. (iv) Hierarchical clusterings are done for the pondered Euclidean distance, the *kNN *and zero replacement methods. 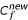 clusters are selected to characterize the new co-association of the genes. From this last step, the Conserved Pairs Proportion (*CPP*) is computed (see Methods).

Figure 1 describes the four steps of the analysis. (i) *Definition of a complete datafile*: We selected from literature a set of microarray data. This set was filtered to retain only genes without MVs. (ii) *Construction of reference gene clusters*: A gene clustering was performed by different HC algorithms (*algo*). Seven types of algorithms were used (see section Methods). For each analysis, we defined a number *K*^*algo *^of gene clusters, representing the reference clusters. (iii) *Generation of a datafile with MVs*: MVs were randomly inserted with a fixed rate *τ*. This rate correspond to the percentage of genes with 1 missing value. So, we defined data with MVs. (iv) *Analysis of the newly generated expression data: *We carry out a data processing similar to step (ii), with the construction of dendograms for each algorithm (*algo*) and the definition of the *K*^*algo *^new clusters in the different experiments (no replacement of MVs was carried out). Finally, we assessed the stability of the gene clustering by calculating an index measuring the percentage of conserved genes between the clusters of the reference set and the generated set.

Moreover, two other experiments were (v) designed and (vi) evaluated: clustering with MVs estimated by the *kNN *approach, and clustering with MVs replaced by zero.

### Experimental sets

As we work on the impact of MVs in gene clustering, we need at first biological datasets without MVs. These sets have been extracted from Ogawa set [26] (noted OS) and Gasch set [25] (noted GS). OS and GS have been chosen because they contain few MVs and, after filtering the number of genes remains important, (*ca. *6000). The original Ogawa set contained 6013 genes with 230 genes having MVs. The elimination of the genes with MVs (i.e. 3.8% of the genes) leads to a set with 5783 genes. For the GS, the number of MVs is more important and some experimental conditions have more than 50% of MVs. So we have limited the final number of selected experimental conditions from 178 to 42 (see section Methods), it allows to conserve 5843 genes, i.e. only 310 genes are not analyzed, representing 5.0% of all the genes.

Moreover, we have defined two smaller sets, GS_H2O2 _and GS_HEAT_, from GS corresponding respectively to H_2_O_2 _and heat shock experimental conditions.

To assess the influence of size of the datasets and the number of observations (genes), we have generated smaller sets corresponding to a ratio 1/*n *(*n *= 2, 3, ..., 7) of the initial OS, GS, GS_H2O2 _and GS_HEAT _gene content (see section Methods).

### Example of clustering disturbance caused by missing values introduction

**Figure 2:**
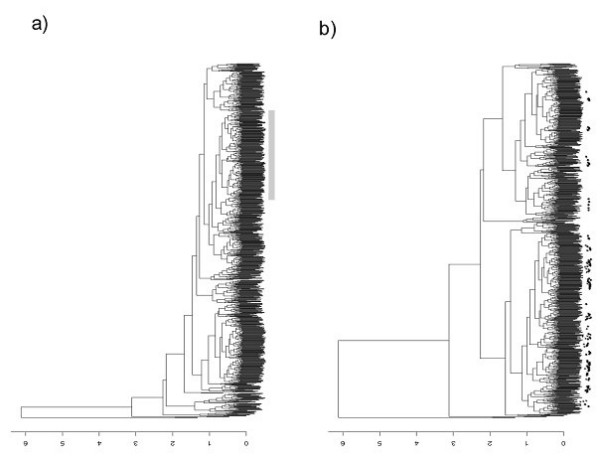
**Example of clustering disturbance caused by missing values introduction. **Hierarchical clustering dendograms from the same set for the complete-linkage method with (a) no MVs and (b) MVs randomly introduced with a rate of *τ *= 1.0%. The genes from one cluster noted by a grey bar in (a) and are shown by stars (*) in (b).

Figure 2 gives an example of significant clustering disturbance caused by the MVs. Figures 2a and 2b show the dendograms obtained with the complete-linkage hierarchical clustering of the gene set before and after introducing 1% of MVs. Surprisingly, the genes belonging to one cluster in the reference dataset are reallocated in several clusters after this slight data transformation.

### Number of clusters

To perform the comparison of the gene clustering by HC, the numbers of clusters according to every type of hierarchical clustering algorithms have to be defined. Hence we defined the number of clusters,*K*^*algo*^, for each clustering algorithm (*algo*). The rule consists in determining *K*^*algo *^clusters as the 10 most important clusters correspond to 80% of the genes of the dataset (see section Methods). The results for OS are shown in Table 2 (column 1) for each clustering methods. As expected, we observe a correlation with the type of algorithm used. The number of clusters is lower for the well-balanced tree generally obtained by the Ward and complete-linkage methods, e.g. 19 and 36 clusters respectively, compared to those providing by the other methods. For instance, the single-and centroid-linkage methods lead to the definition of 175 clusters.

### Index "Conserved Pairs Proportion (CPP)"

**Figure 3:**
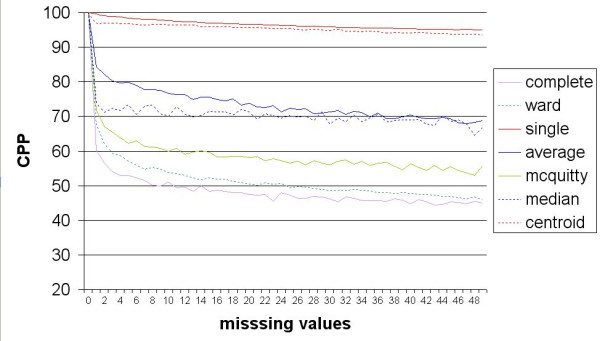
**Variation of the Conserved Pairs Proportion (*CPP*). **The mean *CPP *values of the OS (1/7) are given in function of the percentage of MVs for the seven aggregation methods (complete-linkage, Ward minimum variance, single-linkage, average-linkage, mcquitty, centroid-linkage and median-linkage). 200 independent simulations have been done per rate (*τ*) of MVs.

The clusters defined from the reference data and the data with MVs are compared using *Conserved Pairs Proportion (CPP) index *which corresponds to the percentage of genes found associated in the reference clusters and found again associated in the clusters generated from the data with MVs. Figure 3 summarizes the results about the influence of MVs on the hierarchical clustering. The given results were computed on the (1/7) subset from OS using the seven aggregative algorithms. The metric used is the Euclidean distance. We observe that (i) the single-and the centroid-linkage methods show a low *CPP *decrease, the *CPP *values are always greater than 95%, (ii) the average-and median-linkage methods are within the range [65%; 80%] and (iii) the mcquitty, Ward and complete-linkage methods show the most striking loss. We observe a drastic loss of the clustering stability since *τ *= 1% of MVs. For instance, with 5% of genes with MVs, *i.e*. 40 missing data, the mcquitty, Ward and complete-linkage methods have a *CPP *of 62%, 57% and 52%, respectively. Beyond a rate of *τ *equal to 10%, the decrease becomes lower.

Similar results are observed for all the sets OS, GS, GS_H2O2 _and GS_HEAT _and all the generated sets from 1/2 to 1/7. These last results show that the quality of the gene clustering is not disturbed by the reduction of the number of genes.

It must be clearly noted that to limit the effect of the topology of each algorithm, we have fixed that the 10 most populated clusters must represent 80% of the genes. The number of the most populated clusters is fixed at 10 due to the Ward linkage method that gives a very limited number of clusters. Then, the percentage of genes belonging to the 10 most populated clusters have been tested ranging from 70% to 90% (data not shown). For example with a percentage equal to 90%, the *CPP *values of single-and the centroid-linkage methods remain too stable to observe a clear decrease as seen in Figure 3. The choice of 80% allows to analyze the precise decrease of the different *CPP *values and to compare the different aggregation methods.

### *k*-Nearest Neighbor (*kNN*)

The *kNN *method has been described by Troyanskaya and co-workers [18] for the MVs in microarray data. The *kNN *approach goal is to compute the expected value of a missing value from the *k *nearest vectors without a missing value. As no theoretical approach exists to define the optimal *k *values (*k*_*opt*_), we have assessed every value of *k *within the range 1 to 100 and, selected the *k*_*opt *_value as the *k *value which has the minimal error rate. Table 3 shows the *k*_*opt *_values obtained for the four sets used in this study and their corresponding error rates. The *k*_*opt *_values are lower in OS subsets showing a low number of genes. The *k*_*opt *_values of GS_H2O2 _are within the range 11 to 17. The GS and GS_HEAT _sets exhibit more important variations within the range 8 to 28. The error rate decreases slightly according to the number of genes, but these variations are not significant. Nevertheless, this poor correlation may be due to the subsets composition. Indeed, they keep an equivalent number of clusters with a smaller number of genes per cluster. In the same way, it may simply be due to the *k*_*opt *_variation.

**Figure 4:**
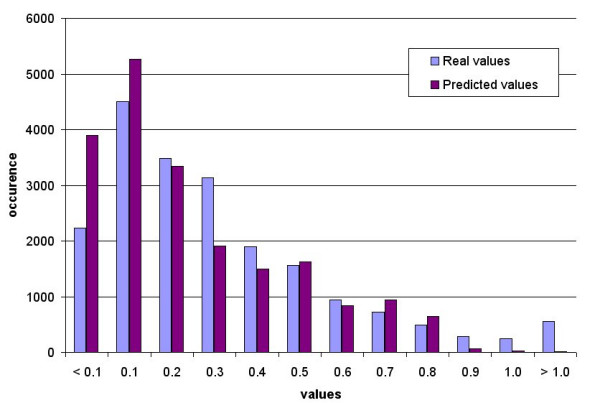
**Comparison of real and *kNN *predicted values distribution. **Histograms of the real values of the OS (1/7) (black bars) and the predicted values using *kNN *method with *k*_*opt *_= 14 (grey bars) are shown. The values are shown in absolute value. All the values superior to 1.0 (respectively inferior to 0.1) have been pooled in the '> 1.0' group (respectively '< 0.1').

Figure 4 shows that the *kNN *method gives worse prediction of the extreme values than the values close to zero. The real data distribution follows approximately a normal distribution and the *kNN *approximation leads to a reduction of the standard deviation of this distribution. So, the prediction of the extreme values increases the global error rate implying a higher *k*_*opt *_to reduce this effect. For instance, in the OS sets, we observe that the values within the range [-1.0; 1.0] are approximated with a mean error rate within the range [0.12; 0.14]. Conversely the values more than 1.5 or less than -1.5 are approximated with a mean error rate superior to 1.8. The misestimating of extreme values has an impact on the clustering. One can notice that the unweighted *kNN *(mean of the *k *observations) exhibits worse results compared to the weighted *kNN *used in this study (data not shown).

### Improvements of CPP with *kNN *approach and zero-value replacements

The *CPP *was computed for the seven agglomeration methods. We have compared the HC results obtained with the reference sets and the generated sets without replacement of MVs, with *kNN *replacement or with *zero-value *replacement methods. Table 2a shows that the *kNN *and *zero-value *replacements both improved the mean *CPP *whatever the clustering method used, except for the Ward method with the zero value replacement. The *kNN *approach is the most relevant method to replace MVs. In 55.2% to 66.3% of the simulations, *kNN *is better and globally gives a mean increase of the *CPP *within the range [0.7; 2.1]. The centroid-and single-linkage methods have better increase in 74.8% and 99.3% of the simulations respectively due to their particular topologies. The zero value replacement is clearly less efficient.

Nevertheless, as a slight variation can displace one gene into a close cluster, we have characterized another index named *CPP*_*f *_to consider the *f *closest clusters of the selected cluster. This index is similar to the previous one and takes into account that the genes may be relocated in close clusters (see section Methods). It allows the evaluation of the topology conservation. We used *f *= 5. We observed that the co-associated genes in the reference sets are often displaced to close clusters in the simulated sets. As observed for the *CPP*, the *kNN *approach improves the *CPP*_*f *_for all the clustering methods in 51.9% to 60.2% of the simulations within the range [0.2; 1.5]. Due to their high initial *CPP *values (97.7% and 98.8%), single-and centroid-linkage methods do not have a gain as previously observed for the *CPP*.

Similar results are obtained with the other sets with slight variations. For example, GS_H2O2 _have *CPP *and *CPP*_*f *_close to the one of OS. Conversely, the *CPP *and *CPP*_*f *_of GS_HEAT _are better than the ones of OS and GS_H2O2 _for the complete-linkage and Ward methods, but lower for the others. The GS set has higher *CPP *and *CPP*_*f *_for single-likage to mcquitty method due to a lower influence of the MVs in a vector with a higher number of experimental conditions. Nevertheless, the complete-linkage and Ward linkage still remain at a very low *CPP *(close to 50%).

Moreover, we have tested the influence of the number of MVs per gene by introducing more than one missing value per gene. We obtained similar results showing less than 0.2% of variations of the *CPP *values. In addition we have tested the consequences of using *k *values different of *k*_*opt *_values in the range [*k*_*opt*_-10 ; *k*_*opt*_+10] and we observed a decrease of *CPP *within the range [1%; 5%] (data not shown).

### Extreme values

We have followed the same methodology to analyze the extreme values, *i.e*. values superior to 1.5 and lower than -1.5. Table 2b summarizes the results of the OS (1/7). The *CPP *values are superior to the *CPP *values obtained previously, because only the genes with important variations have MVs and are members of small clusters. We observe that MVs replacement has little effect. Indeed, the *kNN *and zero – value replacement cannot restore a correct distribution (cf. Figure 4). However, the *CPP*_*f *_shows that the *kNN *is better than the replacement by zero, allowing a better topology preservation. Same results are observed for the other sets (data not shown).

## Conclusions

MVs are a common trait of microarrays experiments. Few works had been reported about MVs replacements [18-20] and none analyse their influence in the clustering of microarrays data. In our study, we showed that MVs significantly biased the hierarchical clustering. In addition, we observed that the effects of MVs are correlated to the chosen clustering method. The single linkage-method is the most stable due to the building of cluster of large size and numerous small clusters and singletons. At the opposite, the Ward and complete-linkage methods create well distributed population of clusters inducing a higher sensitivity to MVs. The topology of the dendogram is highly disturbed by transferring genes in distant clusters.

We showed that the *kNN *replacement method was the most efficient approach to compensate the MVs effects compared to the classical replacement by zero. The *k*_*opt *_depends on the sample size. It is important to keep in mind that the MVs corresponding to extreme values are difficult to estimate with the *kNN *method. The impact of their approximation upon the clustering is significant. Hence, new approaches like the Bayesian Principal Component Analysis (BPCA) may overcome this problem. In a recent work Liu and co-workers suggest to potentially eliminate the incomplete series of data by using robust Singular Values Decomposition [29]. In addition, our work showed clearly the need of evaluation of the data quality and statistical measurements as noted by Tilstone [30].

Contrary to Yeung and co-workers [14] and Gibbons and Roth [15], we have defined for each type of hierarchical clustering algorithm (*algo*) a specific number of clusters (*K*^*algo*^). This point is one of the main difficulties noted by Yeung and co-workers [31] to evaluate the clustering methods as the topology generated are different. The comparison of the different aggregative clustering algorithms remains constrained by the topology (e.g. 175 clusters defined in our study for single-linkage compared to 19 clusters for Ward method). All these results are in accordance with the results of Nikkilä and co-workers [32] which show a hieratic problem of topology preservation in hierarchical clustering. Recent methods like SOTA [33,34] or Growing SOM (Self-Organizing Maps) [35] have combined a hierarchical clustering visualization with the preservation of the topology allowed by the SOM. Our future works will address the definition of a most robust clustering method.

## Methods

### Data sets

We used 8 public data sets from the SMD database ([36]; see Table 1). Two sets were used for a thorough analysis. The first one (Ogawa set) was initially composed of *N *= 6013 genes and *n *= 8 experimental conditions about the phosphate accumulation and the polyphosphate metabolism of the yeast *Saccharomyces cerevisiae *[26]. The second one corresponds to various environmental stress responses in *S. cerevisiae *[25]. This set (Gasch set) contains *N *= 6153 genes and *n *= 178 experimental conditions. Due to the diversity of conditions in this set, we focused on two experimental subsets corresponding to heat shock and H2O2 osmotic shock respectively.

### Data sets refinement: missing values enumeration

To evaluate the incidence of MVs on hierarchical clustering, we built complete datasets without MVs. All the genes containing at least one missing value were eliminated from the Ogawa set (noted OS). The resulting OS set contains *N *= 5783 genes and *n *= 8 experimental conditions. The second set without MVs was taken from Gasch *et al. *and called GS. The experimental conditions (column) containing more than 80 MVs were removed. The resulting GS matrix contains *N *= 5843 genes and *n *= 42 experimental conditions. Two subsets were generated from GS and has been noted GS_HEAT _and GS_H2O2_. They correspond to specific stress conditions as described previously. GS_HEAT _and GS_H2O2_contain respectively *N *= 3643 genes with *n *= 8 experimental conditions and *N *= 5007 genes with *n *= 10 experimental conditions.

To test the influence of the matrix size, *i.e*. the number of genes, we built six smaller sets corresponding to 1/2, 1/3, 1/4, 1/5, 1/6 and 1/7 of OS, GS, GS_HEAT _and GS_H2O2_. To obtain representative subsets, we can not use a random generation which can bias the results. So, we searched for each subset the series of genes which reflect at best all the genes of the complete set. First, the distance matrices between all the genes were computed. Then, we performed an iterative process by: (i) computing the sum of the distances for each possible *t-uplets *(*t=*2 to 7) of the set, (ii) choosing the *t *genes which have the minimal distance, (iii) selecting 1 representative gene upon the *t *selected genes, this gene is chosen as the closest to the barycenter of the cluster, (iv) eliminating from the process the *t *genes. Step (i) to step (iv) are repeated until all the genes are used. All the representative genes constitute the subset (1/*t*). This procedure allows one to reduce the redundancy of similar genes and to maintain approximately a number of gene clusters constant.

### Missing values generation

From the sets without MVs, we introduced a rate *τ *of genes containing one MV (*τ *= 1 to 50.0%), these MVs are randomly drawn. Each random simulation is generated at least 100 times per experiment to ensure a correct sampling.

### Replacement of MVs by the *kNN *method

To fulfill *v*_*i*_, a missing value *i *for a given expression vector **v **(*i.e*. a gene), with the *kNN *method, the *k *vectors **w **corresponding to the *k *most nearest vectors to **v **(without taking the *i*th elements of the **w**-vectors into account) are searched. The missing value *v*_*i *_is then estimated by a weighted value of the *k *retained *w*_*i *_values.

The similar vectors are identified by calculating the Euclidean distance *d *between the vector **v **and every vector **w**. The *k *minimal distances *d*(**v**, **w**) are selected and the estimated value 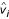 is computed as follow [18,19]:


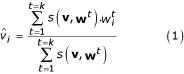


In the weighted *kNN*, *s*(**v**, **w**^*t*^) = 1 / *d*(**v**, **w**^*t*^), this similarity measure *s*(**v**, **w**^*t*^) is deduced from the distance *d*(**v**, **w**^*t*^) between **v **and its neighbors **w**. In equation (1), more a vector **w **close to **v**, more it contributes to the estimation of the missing value. The *kNN *approach has no theoretical criterion to select the optimal *k *value (*k*_*opt*_) [19]. We have estimated for each subset the corresponding *k*_*opt *_using the sets without MVs to ensure a minimal bias in the comparison of the hierarchical clustering results. The determined *k*_*opt *_value is associated with a minimal global error rate as defined by Troyanskaya and co-workers [18].

### Hierarchical Clustering

The hierarchical clustering (HC) algorithm allows the construction of a dendogram of nested clusters based on proximity information [6]. The HC have been performed using the *hclust *package in R software [37]. Seven hierarchical clustering algorithms have been tested: average-linkage, complete-linkage, median-linkage, mcquitty, centroid-linkage, single-linkage and Ward minimum variance [13].

The distance matrix between all the vectors (*i.e*. genes) is calculated by using an external module written in C language. We used the normalized Euclidean distance d* to take account of the MV:


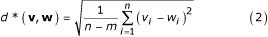


**v **and **w **are two distinct vectors and *m *is the number of MVs between the two vectors. Thus, (*v*_*i *_- *w*_*i*_) is not computed if *v*_*i *_and/or *w*_*i *_is a missing value

### An index for clustering results comparison: Conserved Pairs Proportion (*CPP*)

To assess the influence of missing data rates and different replacement methods into clustering results, we have analysed the co-associated genes of an original dataset (without MVs) compared to these genes location in a set with MVs.

Hence, we realized in a first step the clusterings with the data sets without MV by each aggregative clustering algorithm. The results obtained by these first analyses are denoted *reference clusterings (RC)*. In a second step, we generated MVs in data. The MVs are replaced by using the different replacement methods. Then we performed the hierarchical clustering for each new set. The results obtained by these second analyses are denoted *generated clusterings (GC)*. We compared the resulting clusters defined in *RC *and *GC*. We assessed the divergence by using an index named *Conserved Pair Proportions *(*CPP*). The *CPP *is the maximal proportion of genes belonging to two clusters, one from the *RC *and the other one from the *GC *(cf. Figure 1).

**Figure 5:**
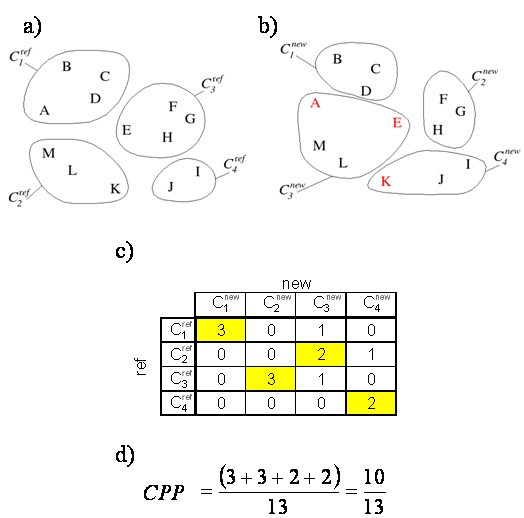
**Example of CPP computation. **a) A hierarchical clustering is performed for a given algorithm and the 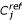 clusters are selected to characterize the original co-association of the genes (with *j *= 4 in the example). b) After the introduction of the MVs, 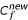 clusters are selected to characterize the new co-association of the genes. c) The number of common genes is computed between the *reference clustering *(ref) and the *generated clustering *(new). In yellow is given the maximum number of common genes allowing the identification of the closest 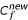 cluster for each 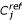 cluster. d) The corresponding *CPP *value is equal to 76.9%

The procedure for computing the index *CPP *is as follow (figure 5 gives an example of *CPP *computation):

i) For each reference clustering based on a given clustering algorithm (*algo*), we defined *K*^*algo*^, the number of clusters. As every type of hierarchical clustering algorithm gives a particular topology, we cannot use the same number of clusters to compare each aggregative method. So, we defined *K*^*algo *^such as its 10 most important cluster must represent 80% of the genes. For this purpose, we defined *K*^*init*^, an important initial number of clusters (equals to 500), and counted the number of occurrences associated to the 10 most populated clusters. Then we diminished *K*^*init *^by one unit and counted again. We stopped the process when the 10 most important clusters represent 80% of the occurrences (*K*^*algo *^= *K*^*init*^). We denote by 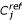 the *j*^*th *^cluster for a given clustering algorithm with *j *= {1, ..., *K*^*algo*^}. The 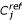 clusters are associated with their corresponding gene list 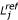.

ii) Three hierarchical clusterings are performed after generating MVs in proportion *τ *in the data, the first one without replacing data – in this case, the normalized Euclidean distance (Eq.2) is used -, the second one after estimating the missing data by the *kNN *method (Eq.1), and the third one after replacing the missing data by zero. For each resulting tree, *K*^*algo *^clusters are defined. The 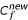 clusters are associated with their corresponding gene list 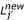, with *j' *= {1, ..., *K*^*algo *^}.

iii) Finally, to estimate the *CPP *index, we searched for each 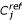 cluster the closest 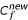 cluster. For each clustering algorithm (*algo*), the corresponding 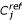 cluster is selected as the maximum number of genes 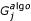 from the gene list 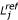 found in 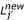. Then, the Conserved Pairs Proportion (CPP) is computed as follow for one simulation:


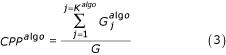


where 
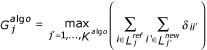
. The term 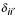 is the Kronecker symbol, *i.e*. it is equal to 1 when the genes *i *and *i' *in the two gene lists are identical, otherwise 0. *G *denotes the total number of genes. This index takes the maximal value 1 when the clusterings *RC *and *GR *are identical.

In addition, a variation of the tree topology may induce a *CPP*-variation. If the remaining genes of a cluster are in the direct neighbour clusters, the use of *CPP *can bias the analysis. Thus, we characterized the *CPP*_*f *_ratio to consider the *f *closest clusters of the 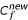 cluster. The computation of *CPP*_*f *_ratio is based on the previous ratio and corresponds to the *f *clusters which are the closest to the winning cluster. From a selected 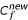, the upper node of the dendrogram is examined. If the number of clusters linked to this node is inferior to *f*, the upper node is selected. This process is performed until the number of clusters is inferior or equals to *f*. The last node tested (*i.e*. with the number of clusters inferior or equal to *f*) is used to compute the *CPP*_*f *_ratio.

## Authors' contributions

AdB conceived of the study and carried out the MVs generation and the hierarchical clustering results. SH worked on the statistical analysis. AM has the initial idea of evaluating the MV influence on clustering, and participated in its design and coordination. All authors read and approved the final manuscript.

## Figures and Tables

**Table 1:** Missing values. Examples of missing value occurrences in different datasets from the yeast *S. cerevisiae *and human microarrays experiments. The year of publication, the studied organism, the number of genes on the DNA chip, the number of experimental conditions, the percentage of MVs and the percentage of genes with missing values are indicated.

Authors	Ref	Organism	Number of genes	Number of experimental conditions	Missing Values percentage (%)	genes with missing values (%)
Bohen *et al*. (2003)	[21]	human	16523	16	7.6	63.6
Sorlie *et al. *(2003)	[22]	human	552	122	6.2	94.7
Garber *et al .*(2001)	[23]	human	918	73	2.4	72.7
Alizadeh *et al *.(2000)	[24]	human	4026	96	5.3	78.8
Gasch *et al. *(2000)	[25]	yeast	6153	178	3.0	87.7
Ogawa *et al. *(2000)	[26]	yeast	6013	8	0.8	3.8
Ferrea *et al. *(1999)	[27]	yeast	5769	4	10.6	30.9
Spellman *et al*. (1998).	[28]	yeast	6178	77	5.9	91.8

**Table 2:** Influence of the replacement method on the Conserved Pairs Proportion (CPP). The numbers of clusters defined per metric are indicated (*K*^*algo*^). The *CPP *and *CPP*_*f *_values are given for the (1/7) subset of Ogawa [26] with missing values introduced (a) in the entire subsets and (b) only in extreme values (values > 1.5 or < -1.5). The *CPP *was computed by using the seven algorithms on the data without replacing the MVs (*MV*), after replacing the MVs by the *kNN *method (*kNN) *with the *k*_*opt *_values (see Table 3), or, after replacing the MVs by zero (*zero*). For *kNN *and *zero *values, the *CPP *and *CPP*_*f *_(with *f *= 5) values are given in (%). The percent of gain for all the simulations is indicated (+/-), i.e. the proportion of simulations with a better *CPP *value than the *CPP *value of *MV*. The values given in this table are the average values for MV in the range [1%; 50%] of genes with one MV per genes.

(a)											
		*CPP*					*CPP*_f_				
		
		*MV*	*kNN*		*zero*		*MV*	*kNN*		*zero*	
	
**Algorithm**	*K*^*algo*^	(%)	(%)	(+/-)	(%)	(+/-)	(%)	(%)	(+/-)	(%)	(+/-)
*single*	175	96.4	98.3	*99.3*	98.2	*99.6*	98.8	*99.1*	*53.7*	99.0	*47.8*
*centroid*	175	95.3	96.0	*74.8*	95.7	*64.1*	97.7	*97.9*	*57.6*	97.8	*53.3*
*average*	83	73.2	75.3	*66.3*	74.8	*62.0*	92.0	*92.5*	*54.4*	93.0	*57.6*
*median*	165	70.1	71.5	*55.2*	72.2	*54.4*	85.2	*86.0*	*52.8*	86.2	*52.1*
*mcquitty*	48	58.2	59.1	*54.5*	58.6	*50.1*	74.9	*75.3*	*51.9*	75.1	*49.5*
*Ward*	19	51.0	52.4	*62.4*	50.7	*49.1*	71.8	*73.3*	*60.2*	71.5	*49.1*
*complete*	36	47.9	49.2	*60.4*	48.4	*51.9*	71.9	*72.7*	*53.7*	72.1	*51.6*
(b)											
		*CPP*					*CPP*_f_				
		
		*MV*	*kNN*		*zero*		*MV*	*kNN*		*zero*	
	
**Algorithm**	*K*^*algo*^	(%)	(%)	(+/-)	(%)	(+/-)	(%)	(%)	(+/-)	(%)	(+/-)

*single*	175	97.9	98.4	*98.5*	98.7	*98.3*	98.8	99.3	*86.9*	99.7	*76.6*
*centroid*	175	96.5	96.7	*64.8*	96.8	*60.6*	98.5	98.5	*55.9*	97.7	*32.8*
*average*	83	81.4	81.0	*45.1*	83.3	*66.1*	93.9	94.5	*57.0*	94.9	*61.3*
*median*	165	75.0	74.2	*45.7*	74.2	*47.9*	87.0	86.5	*50.3*	85.1	*45.4*
*mcquitty*	48	71.6	71.3	*48.1*	71.9	*51.0*	83.1	83.2	*49.6*	84.6	*58.2*
*Ward*	19	63.8	62.4	*42.1*	60.8	*29.9*	83.0	81.1	*37.1*	79.8	*27.0*
*complete*	36	56.5	55.2	*44.5*	56.0	*47.2*	75.7	74.6	*46.1*	74.6	*45.0*

**Table 3:** *kNN *approach: The error rates according to the number of genes. The sets of Ogawa (OS, [26]) and Gasch (GS, GS_HEAT _and GS_H2O2_, [25]) have been cut into subsets representing from (1/2) to (1/7) of the original sets. For each of the subsets, the optimal value for *k *(*k*_*opt*_), i.e. the number of neighbours used in *kNN *approach, has been computed. It was chosen as the number which minimizes the *error rate*. In regards to each subset is given the number of genes (*nb genes*).

	OS			GS			GS_HEAT_			GS_H2O2_		
subsets	*nb genes*	*error rate*	*k*_*opt*_	*nb genes*	*error rate*	*k*_*opt*_	*nb genes*	*error rate*	*k*_*opt*_	*nb genes*	*error rate*	*k*_*opt*_
	
(1/7)	827	0.190	14	815	0.292	18	523	0.183	12	717	0.143	14
(1/6)	968	0.186	20	955	0.276	8	608	0.199	15	837	0.155	11
(1/5)	1159	0.187	20	1141	0.283	23	731	0.195	22	1003	0.155	17
(1/4)	1448	0.180	22	1427	0.270	26	913	0.199	28	1254	0.146	13
(1/3)	1929	0.180	23	1903	0.274	8	1215	0.187	23	1669	0.139	15
(1/2)	2892	0.174	30	2853	0.255	7	1822	0.187	8	2504	0.135	16
set	5783	0.177	39	5705	0.255	8	3643	0.186	10	5007	0.135	17

## References

[B1] DeRisi JL, Iyer VR, Brown PO (1997). Exploring the metabolic and genetic control gene expression on a genomic scale. Science.

[B2] Cho RJ, Mindrinos M, Richards DR, Sapolsky RJ, Anderson M, Drenkard E, Dewdney J, Reuber TL, Stammers M, Federspiel N, Theologis A, Yang WH, Hubbell E, Au M, Chung EY, Lashkari D, Lemieux B, Dean C, Lipshutz RJ, Ausubel FM, Davis RW, Oefner PJ (2003). Genome-wide mapping with biallelic markers in *Arabidopsis thaliana*. Nature Genet.

[B3] Borevitz JO, Liang D, Plouffe D, Chang HS, Zhu T, Weigel D, Berry CC, Winzeler E, Chory J (2003). Large-scale identification of single-feature polymorphisms in complex genomes. Genome Res.

[B4] Liu CH, Ma WL, Shi R, Ou YQ, Zhang B, Zheng WL (2003). Possibility of using DNA chip technology for diagnosis of human papillomavirus. J Biochem Mol Biol.

[B5] Locke DP, Segraves R, Carbone L, Archidiacono N, Albertson DG, Pinkel D, Eichler EE (2003). Large-scale variation among human and great ape genomes determined by array comparative genomic hybridization. Genome Res.

[B6] Everitt B (1974). Cluster Analysis,. Heinemann Educ.

[B7] Hartigan JA, Wong MA (1979). A k-means clustering algorithm,. Applied Statistics.

[B8] Kohonen T (1982). Self-organized formation of topologically correct feature maps. Biol Cybern.

[B9] Kohonen T (2001). Self-Organizing Maps,.

[B10] Mardia KV, Kent JT, Bibby JM (1979). Multivariate Analysis.

[B11] Lee S-I, Batzogolou S (2003). Application of Independent Component Analysis to microarrays. Genome Biology.

[B12] Eisen MB, Spellman PT, Brown PO, Botstein D (1998). Cluster analysis and display of genome-wide expression patterns. Proc Natl Acad Sci USA.

[B13] Quackenbush J (2001). Computational analysis of microarray data. Nature genetics.

[B14] Yeung KY, Haynor DR, Ruzzo WL (2001). Validating clustering for gene expression data. Bioinformatics.

[B15] Gibbons FD, Roth FP (2002). Judging the quality of gene expression-based clustering methods using gene annotation. Genome Res.

[B16] Schuchhardt J, Beule A, Malik A, Wolski H, Eickoff H, Lehrarch H, Herzel H (2000). Normalization strategies for cDNA microarrays. Nucl Acids Res.

[B17] Tu Y, Stolovitzky G, Klein U (2002). Quantitative noise analysis for gene expression microarray experiments. Proc Natl Acad Sci USA.

[B18] Troyanskaya OG, Cantor M, Sherlock G, Brown PO, Hastie T, Tibshirani R, Botstein D, Altman RB (2001). Missing value estimation methods for DNA microarrays. Bioinformatics.

[B19] Oba S, Sato M-A, Takemasa I, Monden M, Matsubara K-I, Ishii S (2003). A Bayesian missing value estimation method for gene expression profile data,. Bioinformatics.

[B20] Zhou X, Wang X, Dougherty ER (2003). Missing-value estimation using linear and non-linear regression with Bayesian gene selection. Bioinformatics.

[B21] Bohen SP, Troyanskaya OG, Alter O, Warnke R, Botstein D, Brown PO, Levy R (2003). Variation in gene expression patterns in follicular lymphoma and the response to rituximab. Proc Natl Acad Sci USA.

[B22] Sorlie T, Tibshirani R, Parker J, Hastie T, Marron JS, Nobel A, Deng S, Johnsen H, Pesich R, Geisler S, Demeter J, Perou CM, Lonning PE, Brown PO, Borresen-Dale AL, Botstein D (2003). Repeated observation of breast tumor subtypes in independent gene expression data sets. Proc Natl Acad Sci USA.

[B23] Garber ME, Troyanskaya OG, Schluens K, Petersen S, Thaesler Z, Pacyna-Gengelbach M, van de Rijn M, Rosen GD, Perou CM, Whyte RI, Altman RB, Brown PO, Botstein D, Petersen I (2001). Diversity of gene expression in adenocarcinoma of the lung. Proc Natl Acad Sci USA.

[B24] Alizadeh AA, Eisen MB, Davis RE, Ma C, Lossos IS, Rosenwald A, Boldrick JC, Sabet H, Tran T, Yu X, Powell JI, Yang L, Marti GE, Moore T, Hudson J, Lu L, Lewis DB, Tibshirani R, Sherlock G, Chan WC, Greiner TC, Weisenburger DD, Armitage JO, Warnke R, Levy R, Wilson W, Grever MR, Byrd JC, Botstein D, Brown PO, Staudt LM (2000). Distinct types of diffuse large B-cell lymphoma identified by gene expression profiling. Nature.

[B25] Gasch AP, Spellman PT, Kao CM, Carmel-Harel O, Eisen MB, Storz G, Botstein D, Brown PO (2000). Genomic expression programs in the response of yeast cells to environmental changes. Mol Biol Cell.

[B26] Ogawa N, DeRisi J, Brown PO (2000). New components of a system for phosphate accumulation and polyphosphate metabolism in *Saccharomyces cerevisiae *revealed by genomic expression analysis. Mol Biol Cell.

[B27] Ferea TL, Botstein D, Brown PO, Rosenzweig RF (1999). Systematic changes in gene expression patterns following adaptive evolution in yeast. Proc Natl Acad Sci USA.

[B28] Spellman PT, Sherlock G, Zhang MQ, Iyer VR, Anders K, Eisen MB, Brown PO, Botstein D, Futcher B (1998). Comprehensive identification of cell cycle-regulated genes of the yeast *Saccharomyces cerevisiae *by microarray hybridization. Mol Biol Cell.

[B29] Liu L, Hawkins DM, Ghosh S, Young SS (2003). Robust singular value decomposition analysis of microarray data. Proc Natl Acad Sci USA.

[B30] Tilstone C (2003). Vital Statistics. Nature.

[B31] Yeung KY, Medvedovic M, Bumgarner RE (2003). Clustering gene-expression data with repeated measurements. Genome Biology.

[B32] Nikkilä J, Törönen P, Kaski S, Venna J, Castren E, Wong G (2002). Analysis and visualization of gene expression data using Self-Organizing Maps. Neural Networks.

[B33] Herrero J, Valencia A, Dopazo J (2001). A hierarchical unsupervised growing neural network for clustering gene expression patterns. Bioinformatics.

[B34] Herrero J, Dopazo J (2002). Combining hierarchical clustering and self-organizing maps for exploratory analysis of gene expression patterns. J Proteome Res.

[B35] Hsu A, Tang SL, Halgamuge S (2003). An unsupervised hierarchical dynamic self-organising approach to cancer class discovery and marker gene identification in microarray data. Bioinformatics.

[B36] Gollub J, Ball CA, Binkley G, Demeter J, Finkelstein DB, Hebert JM, Hernandez-Boussard T, Jin H, Kaloper M, Matese JC, Schroeder M, Brown PO, Botstein D, Sherlock G (2003). The Stanford Microarray Database: data access and quality assessment tools. Nucleic Acids Res.

[B37] Ihaka R, Gentleman R (1996). A Language for Data Analysis and Graphics. J Comput Graph Stat.

